# Adjuvant chemotherapy does not benefit patients with esophageal squamous cell carcinoma treated with definitive chemoradiotherapy

**DOI:** 10.1186/s13014-018-1086-y

**Published:** 2018-08-15

**Authors:** Mingqiu Chen, Minmin Shen, Yu Lin, Pingping Liu, Xiaohong Liu, Xiqing Li, Anchuan Li, Rongqiang Yang, Wei Ni, Xin Zhou, Lurong Zhang, Benhua Xu, Jianhua Lin, Junqiang Chen, Ye Tian

**Affiliations:** 1 Department of Radiation Oncology, the Second Affiliated Hospital of Soochow University, Jiangsu, China; 2 Institute of Radiotherapy & Oncology, Soochow University, Jiangsu, China; 3 Department of Radiation Oncology, Fujian Medical University Union Hospital, No.29,XinQuan Road, GuLou Distric, FuZhou City, FuJian Province China; 4 Fujian Medical University, Fuzhou, China; 5 Department of Radiation Oncology, Fujian Cancer Hospital & Fujian Medical University Cancer Hospital, No. 420, Fumalu Road, JinAn District, FuZhou City, 350014 FuJian Province People’s Republic of China; 6 Cancer and Genetics Research Complex, Department Molecular Genetics and Microbiology, College Medicine, University of Florida, Gainesville, USA; 7 Fujian Key Lab of Individualized Active Immunotherapy and Key Lab of Radiation Biology of Fujian Province Universities, Fuzhou, China; 8 Fujian Platform for Medical Research at First Affiliated Hospital, Fuzhou, China

**Keywords:** Adjuvant chemotherapy, Concurrent chemoradiotherapy, Esophageal squamous cell carcinoma, Survival

## Abstract

**Background:**

The aim of the present study was to assess the efficacy of adjuvant chemotherapy (AC) in patients with esophageal squamous cell carcinoma (ESCC) treated with definitive chemoradiotherapy (CRT).

**Methods:**

The clinical data of patients with ESCC treated with chemoradiotherapy with or without AC were collected and retrospectively reviewed. The overall survival (OS), locoregional failure-free survival (LFFS) and distant failure-free survival (DFFS) rates were analyzed statistically.

**Results:**

A total of 187 patients fulfilled the inclusion criteria, 98 of whom were treated with CRT-alone, while 89 were treated with CRT-AC. Patient characteristics did not significantly differ between the CRT-alone and CRT-AC groups, with the exception of sex and the number of cycles of concurrent chemotherapy. Following CRT, 50 patients achieved complete response (CR), 67 had partial response (PR), 63 patients maintained stable disease (SD) and 7 developed progression of disease (PD). The OS, LFFS and DFFS at 1, 2 and 5 years for the entire cohort were 67.5, 41.4 and 27.2%; 68.7, 57.9 and 52.4%; and 78.5, 68.9 and 63.9%, respectively. The clinical N-stage, M-stage, and short-term response to CRT were identified as significant factors that influenced patient prognosis. No significant differences in OS, LFFS or DFFS were observed between the CRT-alone and CRT-AC groups for the entire cohort and for clinical N-stage, clinical M-stage and short-term response subgroups.

**Conclusions:**

The short-term response to CRT and the tumor clinical stage were significant prognosis factors for patients with ESCC treated with CRT. With current chemotherapy regimens, AC did not improve survival for patients with ESCC treated with CRT. The retrospective nature of the current study serves as a limitation; thus, further clinical trials are required to evaluate the efficacy of AC in patients with ESCC treated with CRT.

## Background

Esophageal cancer is a frequently occurring type of cancer in developing and developed countries [1]. Concurrent chemoradiotherapy (CRT) is considered to be the standard treatment for patients with unresectable esophageal cancer [2]. Several chemotherapeutic drugs and advanced radiotherapy techniques have been applied to treat patients with esophageal cancer in the past decades. The 5-year survival rate of patients with esophageal cancer treated with CRT remains 10–30% [2, 3], although the side effects of treatment were decreased [4]. Uncontrolled tumor growth and local recurrence remain the primary difficulties associated with radiation therapy [5].

For patients who encounter these difficulties, particularly those with uncontrolled tumor growth following CRT, salvage surgery is used and has been declared to improve the survival rate [6]. For patients who cannot undergo or refuse salvage surgery, adjuvant chemotherapy (AC) is often the alternative. However, to date, no large scale clinical trials have been performed to confirm the efficacy of AC following CRT in patients with esophageal cancer, thus no explicit AC guidelines or consensus have been provided in the latest National Comprehensive Cancer Network guidelines [7].

In the current study, the clinical data of patients treated with CRT followed with or without AC at the two eminent specialist cancer hospitals in Fujian Province, China, were collected retrospectively. Patient data were retrospectively analyzed to explore the status of AC in patients with esophageal squamous cell carcinoma (ESCC) treated with CRT.

## Methods

### Patient selection criteria

This retrospective study was approved by Fujian Medical University Union Hospital (No. 2016KY001) and Fujian Province Cancer Hospital (No. K201427) Institutional Review Board. All patients provided written informed consent prior to treatment, and all information was anonymized prior to analysis.

The eligibility and exclusion criteria for the present retrospective study were similar to that reported in our previous study [8]. In brief, the inclusion criteria were as follows: Patients diagnosed with ESCC using histology via esophagogastroduodenoscopy; ≤70 years old; Eastern Cooperative Oncology Group scoring (ECOG) ≤2; clinical stage of TanyNanyM0 or M1 with supraclavicular lymph node metastasis; sufficient pretreatment assessment available to define the clinical stage and to assess the adaptation for treatment (including surgery, chemotherapy and radiotherapy); treated initially with CRT followed with or without AC; no prior salvage surgery performed; and sufficient follow-up data available for short-term treatment response and survival assessment.

The clinical TNM stage was determined according to the 8th American Joint Committee on Cancer (AJCC) TNM staging system [9] based on computed tomography (CT) scan findings analyzed by at least two radiologists. CRT consisted of concurrent chemotherapy (CC) and radiation with three-dimensional conformal radiation therapy (3D-CRT) or intensity modulated radiation therapy (IMRT). CC was defined as chemotherapy which started less than 2 weeks before or 1 week after the initiation of radiotherapy (RT) [10]. AC was defined as chemotherapy initiated at least 2, but less than 6 weeks after the completion of CRT. Whether the AC used was the same drug as CC, or a different drug, was dependent on the short-term response to CRT. Adjustments to the AC and CC time intervals and dose intensities have been reported in our previous study [8].

The targets, including gross tumor volume (GTV), clinical target volume (CTV) and organs at risk (OARs) of radiotherapy, the targets dose and the dose limitations of OARs were defined and adjusted as described in our previous study [8].

### Criteria for toxicity and treatment response

The chemotherapy and acute radiation toxicity were graded using the National Cancer Institute common toxicity criteria (NCI CTC v3.0) [11] and the Radiation Therapy Oncology Group (RTOG) criteria [12], respectively.

The short-term response to CRT was first evaluated on the completion date of CRT and was reassessed after 4–6 weeks. The tumor short-term response to CRT was defined as the clinically complete response (CR), partial response (PR), stable disease (SD) and progression of disease (PD) using the Japanese Classification of Esophageal Cancer guidelines [13]. These response indicators were based on findings from CT scanning and barium esophagography, which were analyzed by two radiologists and confirmed by endoscopic biopsy.

### Surveillance and statistical analysis

The follow-up schedule for patients was as previously reported [8]. In brief, patients were evaluated every 3 months for the first 2 years after CRT, every 6 months for the next 3 years, and then once annually. All patient outcomes were evaluated in March 2018. The primary endpoint was overall survival (OS). The secondary endpoints were locoregional (primary tumor and regional node, including the supraclavicular lymph node) failure-free survival (LFFS) and distant failure-free survival (DFFS). The OS was calculated from the date of diagnosis to the date of mortality or last follow-up. The LFFS and DFFS were defined as the duration between the date of diagnosis to locoregional progression, and distant progression, respectively.

Data were analyzed using SPSS version 18.0 (SPSS, Inc., Chicago, IL, USA). Survival curves were produced using the Kaplan-Meier estimator method and compared with the log-rank test. Multivariable analysis of clinical characteristics (including gender, age, ECOG score, tumor location, clinical TNM stages, the radiotherapy dose of GTV and CTV, regimens and cycles of CC, and short-term tumor response to CRT) was performed using the Cox proportional hazards model. Confidence intervals (CI) represented 95% lower and upper bounds. *P* ≤ 0.05 was considered to indicate a statistically significant difference.

## Results

### Patient characteristics

Between September 1, 2004 and December 31, 2015, a total of 577 patients treated with definitive CRT were reviewed. A total of 193 patients fulfilled the inclusion criteria, of whom 3 patients were administered with single-agent CC and 3 patients succumbed to acute radiation-induced pneumonitis following CRT. These patients were excluded. Data from the remaining 187 patients were collected for analysis. 98 patients were dealt with CRT-alone and 89 were treated with CRT-AC. No significant differences in clinical characteristics were identified between the two groups, with the exception of sex and cycle number of CC (Table 1), which did not influence patient survival in the univariate and multivariate analyses.

**Table 1:** Clinical characteristics of patients

Characteristics	CRT alone	CRT-AC	*X* ^*2*^	*p*
Gender			4.904	0.027
Male	72	77		
Female	26	12		
Median age (y, range)	59 (40–70)	58 (43–70)	0.082	0.265
ECOG scoring			4.342	0.116
0	20	26		
1	76	63		
2	2	0		
Tumor location			0.174	0.982
Cervical	12	12		
Upper	40	34		
Middle	38	36		
Lower	8	7		
Clinical T stage			0,423	0.809
T2	11	12		
T3	46	38		
T4	41	39		
Clinical N stage			3.761	0.153
N0	18	21		
N1	70	65		
N2	10	3		
Clinical M stage			0	0.997
M0	76	69		
M1	22	20		
Clinical stage			2.502	0.475
II	17	20		
III	29	18		
IVA	30	31		
IVB	22	20		
Dose (Gy, range)				
GTV	61.5 (50–66)	61.5 (50–66)	0.023	0.755
CTV	50 (45–54)	50 (45–50.4)	0.081	0.282
Cycles of CC			8.294	0.016
1	34	19		
2	62	61		
3	2	9		
Regimen of CC			0.296	0.586
PF	16	12		
TP	82	77		

*CC* concurrent chemotherapy; *CRT-AC* chemoradiotherapy followed with adjuvant chemotherapy; *CRT* concurrent chemoradiotherapy; *ECOG* Eastern Cooperative Oncology Group scoring; *GTV* gross tumor volume; *CTV* clinical target volume; *PF* platinum plus fluorouracil; *TP* platinum plus taxane; *M1* supraclavicular lymph node metastasis

A median number of 2 (range, 1–3) cycles of CC were administrated to all enrolled patients. The regimens of CC included a dual-agent platinum compound (cisplatin, lobaplatin, nidaplatinum or oxaliplatin) plus fluoropyrimidine (5-fluorouracil or capecitabine; PF) or a platinum compound plus taxane (paclitaxel or docetaxel; TP) [14, 15]. The differences in CC regimens were not significant between the CRT-alone and CRT-AC groups (Table 1).

### Tumor response, failure pattern and survival

Following CRT, 50 (26.7%) patients achieved CR (26 in CRT-alone and 24 in CRT-AC), 67 (35.8%) had PR (37 and 30, respectively), 63 (33.7%) maintained SD (31 and 32, respectively) and 7 (3.7%) developed PD (4 and 3, respectively) (Table 2). The treatment failure patterns are presented in Table 2.

**Table 2:** Tumor response, failure Pattern and survival

	CRT-alone	CRT-AC	Total	p*
Tumor response, n (%)				0.91
CR	26 (26.5)	24 (27.0)	50 (26.7)	
PR	37 (37.8)	30 (33.7)	67 (35.8)	
SD	31 (31.6)	32 (36.0)	63 (33.7)	
PD	4 (4.1)	3 (3.3)	7 (3.7)	
Pattern of failure, n (%)				0.399
Locoregional alone	30(16.1)	35(18.7)	65(34.8)	
Locoregional and distant	6 (3.2)	2 (1.1)	8(4.3)	
Distant alone	26 (13.9)	21 (11.2)	47(25.1)	
1, 2,5 y survival rates (%)				
OS	66.7, 39.1, 27.6	75.3, 47.1, 26.9	67.5,41.4, 27.2	0.732
LFFS	70.4, 57.2, 55.1	69.7, 58.6,49.8	68.7,57.9, 52.4	0.876
DFFS	74.1, 65.3, 63	83.3, 73, 65.4	78.5, 68.9, 63.9	0.200

*: *p* value between CRT-alone and CRT-AC

**Fig. 1:**
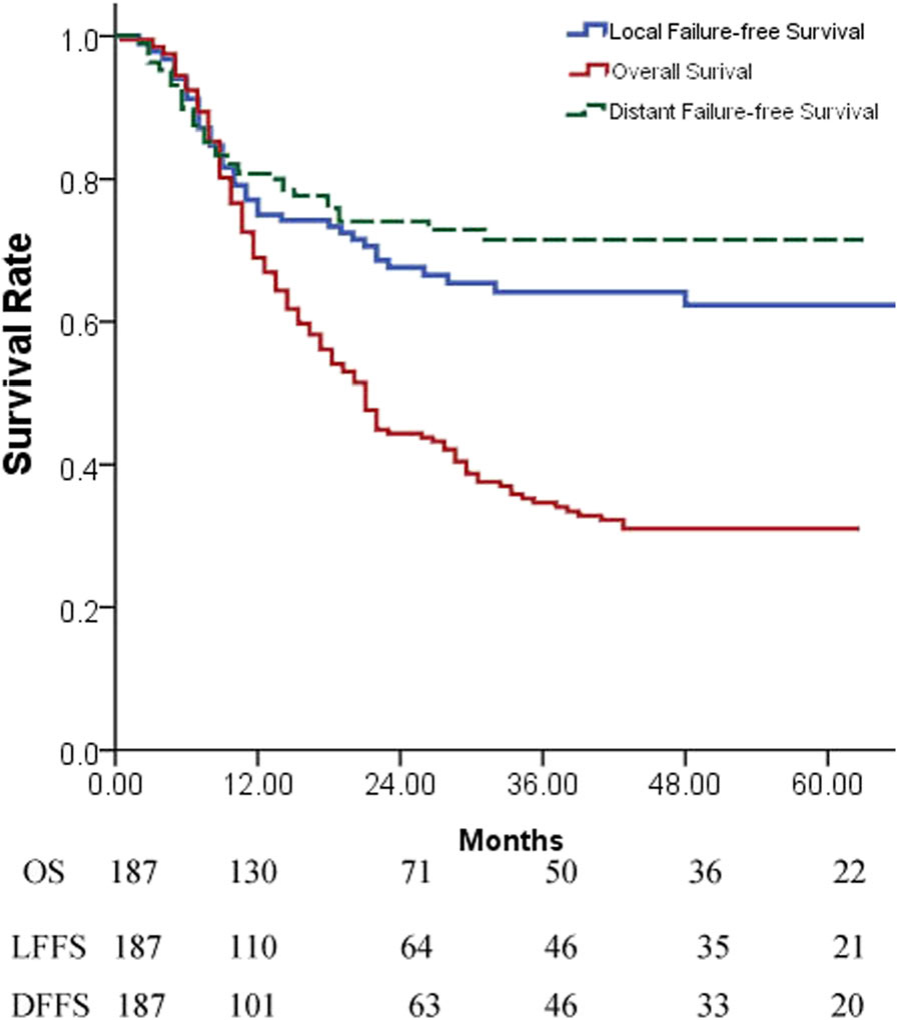
The OS,LFFS,DFFS in the entire cohort of patients

**Fig. 2:**
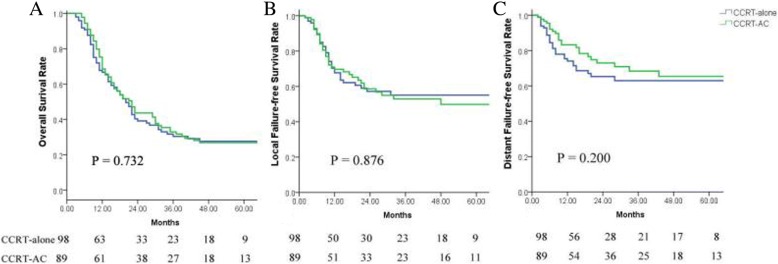
The OS (**a**), LFFS (**b**), DFFS (**c**) between CRT alone and CRT-AC in the entire cohort of patients

At the last follow-up, 54 patients remained alive, 133 patients succumbed. Of these, 114 patients succumbed to the disease (61 for locoregional recurrence, 46 for distant metastasis, 7 for both locoregional and distant) and 19 patients succumbed for unknown reasons. The median follow-up time in the current study was 20 months (range, 3–124 months). The OS, LFFS and DFFS at 1, 2 and 5 years for the entire cohort were 67.5, 41.4 and 27.2%; 68.7, 57.9 and 52.4%; and 78.5, 68.9 and 63.9%, respectively. No significant differences in OS, LFFS or DFFS were observed between the CRT-alone and CRT-AC groups (Table 2; Figs. 1 and 2).

**Fig. 3:**
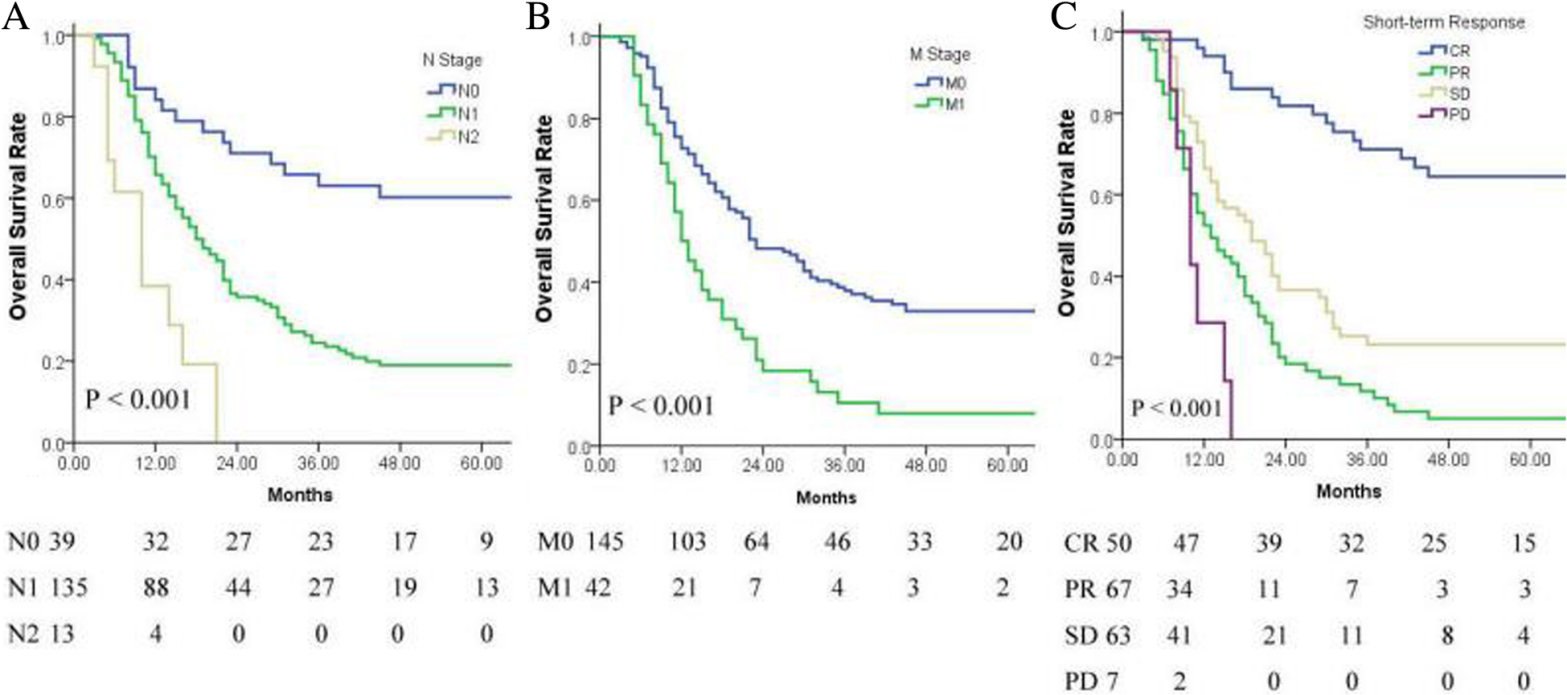
The OS in various subgroups of independent significant factors N-stage (**a**), M-stage (**b**) and short-term response (**c**)

Univariate and multivariate analyses indicated that clinical N-stage, clinical M-stage and short-term response to CRT were significant factors that influenced OS (Table 3 and Fig. 3). Clinical N-stage and short-term response to CRT were the factors to significantly influence LFFS, whereas clinical N-stage and M-stage were the factors to significantly influence DFFS (Table 3).

**Table 3:** Prognostic factors by univariate and multivariate analyses

	OS		LFFS		DFFS		OS		LFFS		DFFS	
Prognostic factors	*p*	HR (95.0% CI)	*p*	HR (95.0% CI)	*p*	HR (95.0% CI)	*p*	HR (95.0% CI)	*p*	HR (95.0% CI)	*p*	HR (95.0% CI)
Gender	0.38	0.817 (0.521–1.281)	0.37	0.75 (0.406–1.402)	0.96	0.984 (0.508–1.907)						
Age	0.70	1.005 (0.980–1.031)	0.03	0.97 (0.934–0.997)	0.03	1.049 (1.005–1.094)						
ECOG	0.60	0.904 (0.622–1.313)	0.51	0.85 (0.521–1.385)	0.68	1.133 (0.627–2.050)						
Tumor location	0.05	0.814 (0.661–1.001)	0.01	0.69 (0.518–0.905)	0.64	0.927 (0.671–1.279)						
Clinical T stage	0.19	1.191 (0.919–1.543)	0.98	1.00 (0.714–1.414)	0.47	1.158 (0.775–1.730)						
Clinical N stage	< 0.01	2.996(2.037–4.408)	0.00	2.68 (1.671–4.289)	< 0.01	2.495 (1.440–4.324)	< 0.01	2.465 (1.612–3.769)	< 0.01	2.293 (1.384–3.798)	< 0.01	2.418 (1.345–4.346)
Clinical M stage	< 0.01	2.142(1.463–3.136)	0.02	1.83 (1.094–3.046)	< 0.01	2.463 (1.391–4.362)	< 0.01	1.856 (1.268–2.716)			< 0.01	2.424 (1.358–4.238)
Clinical TNM stage	< 0.01	1.402(1.181–1.665)	0.03	1.29 (1.023–1.616)	< 0.01	1.487 (1.133–1.953)						
Cycles of CC	0.95	0.990(0.719–1.365)	0.83	1.05 (0.682–1.608)	0.20	0.725 (0.442–1.187)						
Regimen of CC	0.07	0.646(0.405–1.030)	0.87	0.94 (0.485–1.481)	0.63	1.235 (0.529–2.884)						
Dose of GTV	0.51	1.000(0.999–1.000)	0.06	1.00 (0.999–1.000)	0.81	1.000 (0.999–1.001)						
Dose of CTV	0.18	1.001(0.999–1.003)	0.78	1.00 (0.999–1.002)	0.70	1.000 (0.998–1.002)						
Tumor response to CRT	< 0.01	1.691(1.389–2.060)	< 0.01	1.71 (1.315–2.228)	0.44	1.127 (0.834–1.524)	< 0.01	1.548 (1.262–1.899)	< 0.01	1.532 (1.162–2.021)		
AC	0.74	0.971(0.819–1.152)	0.88	1.02 (0.809–1.281)	0.21	0.841 (0.643–1.100)						

*CC* concurrent chemotherapy; *GTV* gross tumor volume; *CTV* clinical target volume; *CRT* concurrent chemoradiotherapy; *AC* adjuvant chemotherapy

Patients who achieved CR had improved survival rates compared with non-CR (PR, SD and PD) in terms of OS, LFFS and DFFS. Furthermore, the survival rates were not significantly different among non-CR patients. Notably, PR patients exhibited worse OS compared with SD patients; however, this difference was not statistically significant (Fig. 3C). This observation may be explained by PR patients exhibiting worse clinical N and M stages compared with SD patients, which were both identified as significant prognostic factors.

### Adjuvant chemotherapy and survival

A median number of 2 (range, 1–4) cycles of AC were performed in patients who received AC. The regimens of AC and CC were the same in CR and PR patients. Only 5 SD patients had their treatment regimen changed from CC to AC, whereas all 3 PD patients in the AC group had their treatment regimen changed. As there are so few effective chemotherapy drugs for ESCC, if a different drug was required for AC, the drug would be changed from PF to TP or vice versa, with different compounds for T, F or P.

To identify patients who may benefit from AC, we conducted further analysis among various subgroups of patients based on different significant prognostic factors, including clinical N-stage (N0, 1 and 2), clinical M-stage (M0 and M1) and short-term response to CRT (CR, PR, SD and PD). No significant differences in survival (OS, LFFS and DFFS) between patients treated with CRT-AC or CRT-alone were observed for any subgroups (Table 4).

**Table 4:** Efficacy of AC in different subgroups

		1,2,5-y OS			1,2,5-y LFFS			1,2,5-y DFFS		
		CRT-alone	CRT-AC	*p*	CRT-alone	CRT-AC	*p*	CRT-alone	CRT-AC	*p*
Clinical N stage	N0	76.5, 58.8, 52.3	95.0, 81.0, 66.7	0.297	69.3, 69.3, 69.3	95.2, 95.2, 89.6	0.105	94.4, 87.2, 87.2	95.2, 89.6, 89.6	0.777
	N1	68.2, 37.9, 23.5	79.0, 33.6, 14.4	0.324	69.7, 58.8, 55.3	62.6, 45.7, 31.3	0.131	68.9, 60.7, 57.3	78.5, 68.3, 53.4	0.377
	N2	40.0, 26.7, 0.0	33.0, 0.0, 0.0	0.785	51.9, 0.0, .0.0	33.3, 33.3, 33.3	0.877	80.0, 53.3, 53.3	0.0, 0.0, 0.0	0.942
Clinical M stage	M0	71.7, 45.8, 35.5	79.7,57.0, 31.0	0.880	70.0, 61.0, 61.0	70.6, 63.5, 57.1	0.860	79.5, 70.3, 67.5	89.6, 79.4, 79.4	0.191
	M1	50.0, 18.2, 4.5	50.0, 18.7, 12.5	0.936	60.6, 44.2, 29.5	67.3, 26.9, 0.0	0.692	52.6, 46.1, 46.1	59.2, 47.4, 47.4	0.792
Clinical TNM stage	II	75.3, 43.9,43.9	95.0, 80.0, 55.0	0.208	72.7, 58.2, 58.2	90.0, 90.0, 84.4	0.079	81.6, 74.2, 74.2	95.0, 95.0, 87.1	0.195
	III	71.7, 37.9, 33.2	61.1, 38.9, 16.7	0.378	68.8, 51.6, 51.6	59.3, 37.6, 37.6	0.549	61.4, 62.2, 62.2	87.7, 68.2, 56.8	0.598
	IVA	69.7, 54.9, 33.8	67.7, 38.7, 23.5	0.651	77.8, 72.9, 72.9	64.2, 59.9, 47.2	0.120	86.7, 76.6, 69.7	86.6, 71.9, 63.9	0.878
short-term response	CR	88.5,76.5,58.4	100, 87.5,78.0	0.274	88.0, 79.4, 74.1	95.8, 91.3, 84.2	0.276	84.4, 76.2, 71.1	95.8, 83.3, 74.8	0.590
	PR	46.1,17.1,12	60.0, 20.0, 0.0	0.998	55.4, 50.8, 50.8	68.2, 52.8, 33.0	0.954	60.6, 48.6, 48.6	67.9, 55.7, 55.7	0.470
	SD	77.4, 36.1,24	56.3, 37.2, 22.3	0.616	65.7, 48.3, 48.3	50.8, 36.9, 31.6	0.277	80.3, 72.3, 72.3	92.8, 86.2, 77.6	0.117
	PD	25.0, 0.0, 0.0	33.3, 0.0, 0.0	0.704	33.3, 33.3, 33.3	66.7, 66.7, 66.7	0.642	75.0, 75.0, 75.0	0.0, 0.0, 0.0	0.247

*M1*: supraclavicular lymph node metastasis

## Discussion

Hishikawa et al. [16] had firstly conducted a randomized clinical trial to evaluate the benefits of AC in patients with ESCC treated with RT in Japan in 1991. In this study, patients with unresectable esophageal cancer were randomized and treated with RT followed with or without AC. The results indicated that, compared with RT-alone, AC did not improve the survival of patients treated with RT-AC. However, the biggest limitation of this research was that patients were treated with RT-alone, which had been verified to have poorer efficacy compared with CRT in numerous clinical trials and might impact the benefits of AC [17].

To the best of our knowledge, no studies have been performed to specifically investigate the effects of AC following CRT without prior salvage surgery, and the current study took the lead in discussing the efficacy of AC in patients with ESCC treated with CRT. Unfortunately, similarly to Hishikawa’s results as manifested in this study, compared with CRT-alone, AC following CRT had not demonstrated significant survival benefits in patients with ESCC. Insufficient survival benefits of AC were usually owned to patient intolerance to intensive AC resulting from the acute toxicity of CRT [18]. Therefore, combinations of newer and more tolerable chemotherapeutic agents before, rather than after, CRT to improve the efficacy of radiotherapy should be considered [19].

It is well established that the initial stage of cancer is the most important in determining the prognosis and treatment plan. The later the staging, the worse the prognosis of patients, and this often means that patients require more intense treatments, such as chemotherapy, in order to improve survival. To identify whether intense treatments such as AC may improve the survival of patients at different tumor stages, we performed a stratified analysis of different staging factors, including clinical N and M stages, that were recognized as independent prognosis factors in the present study. However, our results revealed no significant differences in survival for subgroups stratified by N-stage (N0–2) or M-stage (M0 and M1) treated with or without AC. Noteworthy, patients with N2 or M1 (supraclavicular lymph node metastasis) stage who were regarded as prone to develop distant organ metastases and initially expected to benefit from AC, failed to demonstrate a survival advantage with the use of AC in the current study. This indicated that more intensive chemotherapy regimens to eradicate occult metastases were urgently needed to improve patient outcomes [20].

As demonstrated here and in previous studies [8, 21], the short-term response to CRT is a powerful predictor of survival in patients with ESCC treated with CRT. Patients who achieve CR exhibit improved survival rates compared with non-CR patients in terms of OS, LFFS and DFFS. However, no significant differences in survival rates were observed among non-CR patients (PR vs. SD). These results are consistent with the data from our previous single-center study [8], suggesting that aggressive treatment such as escalating irradiation-dose of tumor by modern radiation techniques to obtain better short-term response should be executed [22].

In preceding studies, the benefits of AC in ESCC patients treated with trimodal therapy (neoadjuvant CRT, surgery and AC, TMT) have varied depending on the short-term response to CRT, and even within short-term response subgroups, the results have been inconsistent. Tam et al.*....* reported that AC in TMT improved the OS in patients with PR to neoadjuvant CRT, but not in complete responders and non-responders [23]. While, Kim et al [24] found that AC only improved the OS in patients with gross residual disease, but not patients with CR or macroscopic residual disease. In contrast, a novel study from Saeed et al indicated that AC did not improve survival in patients treated with TMT regardless of the response to neoadjuvant CRT [25]. However, no studies had been performed to identify which short-term response subgroups of patients with ESCC would benefit from AC following CRT without surgery. Disappointingly, compared with CRT-alone, AC had not demonstrated prolongation of survival in various response subgroups of patients in the current study. The discrepancy between our results and other studies indicates the need for further prospective randomized clinical trials to determine whether certain subgroups of patients who might potentially benefit from AC can be identified based on short-term response to CRT.

There were certain limitations to the present study, such as the retrospective design, the inadequate intensity of chemotherapy and unified chemotherapy regimens, the lack of change with adjuvant CC in patients with SD response to CRT, the lower number of AC cycles, and the suboptimal assessment of short-term response by CT scan. Due to these limitations, the results of our investigation must be interpreted with caution. In addition, the role of AC as a possible palliative therapy to relieve dysphagia, which is the most common symptom of esophageal cancer seriously affecting the quality of life of the patients [14], has not been discussed in the current study.

## Conclusions

The short-term response to CRT and the tumor clinical stage were identified as significant prognosis factors for patients with ESCC. With the current chemotherapy regimens, AC did not provide any significant improvements in patient survival following CRT. The retrospective nature of the current study is a limitation; thus, further clinical trials are required to evaluate the efficacy of AC in patients with ESCC treated with CRT.

## Abbreviations

3D-CRT: three-dimensional conformal radiation therapy
AC: adjuvant chemotherapy
AJCC: American Joint Committee on Cancer
CC: concurrent chemotherapy
CI: Confidence intervals
CR: complete response
CRT: Concurrent chemoradiotherapy
CT: computed tomography
CTV: clinical target volume
DFFS: distant failure-free survival
ECOG: Eastern Cooperative Oncology Group
ESCC: esophageal squamous cell carcinoma
GTV: gross tumor volume
IMRT: intensity modulated radiation therapy
LFFS: Locoregional failure-free survival
NCI CTC: National Cancer Institute common toxicity criteria
OAR: organ at risk
OS: overall survival
PD: progression of disease
PF: platinum compound plus fluoropyrimidine
PR: partial response
RT: radiotherapy
RTOG: Radiation Therapy Oncology Group
SD: stable disease
TP: platinum compound plus taxane
